# Neurodiversity, Networks, and Narratives: Exploring Intimacy and Expressive Freedom in the Time of Covid-19

**DOI:** 10.17645/si.v11i1.5737

**Published:** 2023-01-17

**Authors:** Kerri Betts, Louise Creechan, Rosemarie Cawkwell, Isabelle Finn-Kelcey, C.J. Griffin, Alice Hagopian, David Hartley, Marie Adrienne R. Manalili, Inika Murkumbi, Sarinah O’Donoghue, Cassandra Shanahan, Anna Stenning, Alyssa Hillary Zisk

**Affiliations:** 1 School of Languages, Cultures and Societies, University of Leeds, UK; 2 Department of English Studies, Durham University, UK; 3 Institute for Medical Humanities, Durham University, UK; 4 Independent Researcher, UK; 5 Department of Education, University of Winchester, UK; 6 Department of English and Comparative Literary Studies, University of Warwick, UK; 7 School of Modern Languages, University of St Andrews, UK; 8 Department of Creative Writing, University of Manchester, UK; 9 Division of Language and Communication Science, University College London, UK; 10 Department of Social Anthropology, University of Cambridge, UK; 11 Department of English Literature, University of Aberdeen, UK; 12 Department Literature and Creative Writing, Macquarie University, Australia; 13 School of English, University of Leeds, UK; 14 Department of Neuroscience, University of Rhode Island, USA; 15 AssistiveWare, The Netherlands

**Keywords:** autism, collaboration, narratives, neurodivergence, neurodiversity, online community, self-advocacy, social networks

## Abstract

The Narratives of Neurodiversity Network (NNN) is a neurodivergent academic, creative, and educator collective that came together with allies during the Covid-19 pandemic to create a network centred around emerging narratives about neurodiversity and exploring new ways of learning and socialising. The network focuses on exploring the roles of written, spoken, and visual narratives across cultural locations about neuro-atypical experiences in generating improved agency and self-advocacy for those who have been subject to pathologization through neuro-normativity and intersecting oppression. During the last year, widening access to digital platforms has provided a space to explore these issues outside of traditional academic spaces. We run a monthly “Salon,” our mixed-media “reading, listening, and watching” group, in an effort to find positive representation within contemporary culture. Discussions have moved beyond mimesis and into a consideration of how narrative and storyworlds can question the supposed naturalness of certain ways of being in and perceiving the world. This article interrogates the network’s core principles of nonhierarchical co-production, including the roles of creativity, community, identity, and emancipatory research which were animated by the new techno-social context. We consider the cultural lives of neurodiversity in the West and beyond, including ethical and aesthetic dimensions. We share a faith in the power of storytelling to inform new social identities for neurodivergent people and to inform scientific understandings of atypical cognition. In exploring this, we speak through a porous first-person plural narrator, to unsettle the idea that there is a hegemonic “we” speaking on behalf of all neurodivergent people.

## 1 Introduction: A Note on Neurodiversity, Narrative Diversity, and Method

I’m into neurodiversity, communication, and representation in a lot of ways. There is no one home for *all* my research interests at once but this space holds some interests that don’t live in any of the academic departments I’ve been in or any of the jobs I’ve had. Neurodiversity in the divergent universe? Creative nonfiction about neurodivergent experiences? Yeah, those go *here*.

Narrative is central to this article. It represents both a shared interest that united our network’s members during the Covid‐19 pandemic and an emancipatory means of neurodivergent self‐fashioning. It is also integral to the research for and presentation of this article. When we say “narrative,” we capture how our members use narrative and storytelling to connect, self-advocate, navigate, and engage within an online space. We invite readers to view this article as a meta-discourse, as we place individual members’ words in dialogue with one another and with our overarching themes. We also use “narratives” to place equal value on the varied viewpoints (or narratives) as a reminder of the pluralities and divergences even under a collective (and largely co-produced) piece and to reject the neuronormative primacy of spoken verbal conversation implied in “voices” or “polyphony” (Wood, 2021). The first-person plural “us” and “we” signal our collective positionality. Relatedly, the article is divided into subsections that represent differing relationalities to the concept of narrative, but we acknowledge this article’s narrative might occasionally appear to be non-linear, tangential, or even contradictory. This is calculated and purposeful as we communicate the nuances and variations of expression inherent in a neurodivergent-majority space within the constraints of an academic article and reject the neuronormative expectation of linearity (Yergeau, 2018, p. 19). By uniting diverse perspectives under a collectively written article, we are consciously enacting the key tenet of the larger neurodiversity movement (Kapp, 2020, p. 330) by acknowledging the myriad embodied affective and cognitive differences among humans, which exceeds the currently recognised medical categories of neurological difference due to the dominance of a singular ideal of subjectivity.

We wanted to give all network members the opportunity to contribute during production. To logistically manage to co-write with 317 network members, this article’s main body (text not marked as quotation) was written collaboratively by some co-founders of the Narratives of Neurodiversity Network (NNN) to capture responses to questions members were invited to respond to. Importantly, this invitation was also extended to those drafting the article. This configuration moved us away from the “academic as observer” model, to foreground shared community, and work towards a model of co-production. While the development of our methodology for this research (and later its dissemination) was inspired largely by our efforts to centre the lived experience of our members outside of traditional participatory research models, we acknowledge our debt to previous scholarship that foregrounds neurodivergent co-writing (Bertilsdotter Rosqvist et al., 2019, 2020a; Fletcher-Watson et al., 2019). The nuance within our approach is part of the network’s broader aim to acknowledge and disrupt systemic power imbalances, where those with more educational or cultural capital (here, academics), often appear to be doing “all the work,” even when others are subtly shaping the group (for example, engaging with other members and providing humour, references, interpretations, or passion). We acknowledge this approach is not true co-production, as those who drafted the article maintained a level of editorial privilege as they decided which questions were put to the wider network, which discussion points to focus on, and which responses were quoted. To mitigate this privilege, we invited all network members to respond to the article and suggest edits during drafting. Thus, network members were not greeted with a finished product and the implication that we had already decided how to present their insights. Similarly, all member quotations are attributed anonymously to “one member.” We only identify context when relevant and with permission. All network members who contributed to the article’s formulation or the discussions around its content are named—even if not quoted directly. We also, therefore, follow the alphabetical authorship convention common among many-author papers and when determining relative contributions is impractical (Fernandes & Cortez, 2020) or, in our case, undesirable.

## 2 Narratives of Neurodiversity Network: Beginnings, Aims, and Technology

For many disabled and/or neurodivergent people, the initial months of the pandemic were a highly contradictory period. Many were designated “clinically vulnerable” and subject to stricter lockdown protocols while media discourses on issues, such as vaccination, mask-wearing, the lifting of lockdown legislation, and what kind of people would be more likely to experience a greater adverse reaction to Covid infection, often centred on disabled persons (Imperatore, 2021). Additionally, previously utilised health and social care were often not provided during the pandemic (Flynn & Hatton, 2021). However, alongside these narratives of disempowerment, the necessary shift to remote working meant accommodations that had seemed impossible or impractical prior to the pandemic were suddenly found feasible (Ryan, 2020). Accessible technology and home working and studying became a requirement for the abled majority, and, through this fundamental reconfiguration of labour models, many found these pandemic adaptations enabling. As workspaces became online spaces in this unprecedented moment of lockdown, the possibilities for fostering community, cultural life, and connections with others from across the world seemed more tangible.

In August 2020, while the world was in the throes of the first Covid-19 wave, one of the founders of what is now the NNN sent a tweet looking to develop a network for writers and creatives interested in neurodiversity. To gauge interest, she wrote: “I’d love to hear from other people who are working on autism or neurodiversity and literature or creative writing. I think there are quite a few of us.” “Neurodiversity,” the notion that all brain types are valid and that neurological differences cannot and should not be “corrected” or “cured,” and “neurodivergence,” a term developed by neurodivergent communities to describe themselves, were developed in the late 1990s (Arnold, 2017; Asasumasu, 1999). The term “neurodiversity” is often attributed to Singer (1999), but we wish to acknowledge that the term was emerging simultaneously within online autistic spaces. The field has since expanded, and “neurodiversity” has become a central theme in the work of many writers, theorists, and creatives in the UK and is also gaining some traction around the world (Manalili, 2021). It also denotes an activist position and investment in advancing the equalising agenda of the neurodiversity movement (Walker, 2021). Outside of more formal discourses, neurodiversity is an empowering term used by many neurodivergent people, who contest the idea that their way of being is lesser. Despite the growing popularity and awareness of neurodiversity as a concept, we felt that there was a scarcity of majority neurodivergent spaces where we could share resources and support one another. The series of tweets that followed received engagement from users from a wide variety of backgrounds, disciplines, geographical locations, and neurodiverse positionalities (we include neurotypical allies within our space and our neurodivergent members have a variety of diagnoses, including, but not limited to, autism, ADHD, dyslexia). Significantly, several literary scholars answered the initial call for connections. Together, we realised our interest in neurodiversity was mediated through our engagement with fiction and creative writing. We soon refined this initial observation as we learned narrative’s implicit and liberating role as a vehicle for exploring neurodivergent identity, developing a community united by shared understandings, and enabling self-advocacy.

The subsequent idea was to create some sort of neurodivergent-led communication channel and resource hub where people in these areas could both reach out and provide support and/or solidarity to others. The network began—as many academic networks do—with a Jiscmail list as the Listserv Neurodiversity and Literature. The server became a limiting space, rather than a liberating one, as the longer time scales of monitored email exchanges could not support the rapid influx of new members engaging at their desired scale. Members expressed a desire to forge connections outside the “formal” constraints of email and to interact without “copying in” the entire group, as required by the server. Additionally, Listserv’s firm association with academia was becoming an issue. While anyone with an email address can access Jiscmail lists, these servers are synonymous with higher education institutions. Acknowledging this, many members from outside the academy began with the caveat: “I am not an academic but….” This phraseology signalled that, despite our desire to create a community of interested persons irrespective of formal academic credentials, we had inadvertently created a hierarchy through our choice of a more traditional academic model of online networking. Aided in part by the new technological norms of pandemic working models, we sought online services that supported greater conversational immediacy. We found Zoom an invaluable asset in this sense for our mixed-media reading/listening group, the Salon, and to enable direct conversation. Eventually, we established our network on Discord, an instant message and digital distribution platform that supports a variety of access needs and communication mediums, including asynchronous and instant messaging (including text and voice), video calling, and photo sharing across simultaneously existing channels. Typically, a singular Discord server has many channels, and members may select which discussions they contribute to, which has enabled smaller communities to form within the network of members with specific foci, such as creative writing, academic discussions, and general socialising. One member explained that, since becoming more familiar with the Discord server and its functions, they now find it less demanding than other communicative formats:

I was completely new to Discord at the time when our network migrated to it. I am really not tech savvy, but I got used to it fairly easily and I find it much easier to keep track of than some other formats such as email. It suits me to be able to read a comment and respond in my own time (or not respond).

The alternative pacing of Discord encourages members to respond out of keenness or shared interest rather than obligation. By supporting multiple, coexisting discussions on a vast array of topics, more academic and potentially exclusionary discussions are decentred and positioned as only one of many aspects of the network’s engagement with narratives of neurodiversity.

By situating our interactions in a shared online space, we acknowledge the important history of this model as a site of early autism advocacy and activism in the late 1990s and early 2000s. Our Discord channel is indebted to this legacy of networked communities formed by and for the neurodivergent. Historically, the removal of the need for face-to-face and verbal interactions enabled many neurodivergent individuals to take to online blogging and forum creation as a safer and more comfortable method of expression (Blume, 1997; Davidson, 2008). Most of the contributions to the Autism Self Advocacy Network’s anthology *Loud Hands: Autistic People, Speaking* were taken directly from the blogs of autistic writers such as Julia Bascom, Nick Walker, Remi Yergeau, and Mel Baggs, a practice that indicates the impact that the free expression and comment-based interchange of blogging had on the formulations of the neurodiversity paradigm. Indeed, adapting digital spaces for the needs of an emergent community has become something of a particular talent of the neurodivergent who have been led, in part, by social necessity (Sinclair, 2012). For our network, adopting a somewhat more private forum space on Discord has allowed us to take a digital step away from the current dominance of social media such as Twitter and Facebook. Neurodivergent presence remains strong and useful in these spaces, but often finds itself acutely exposed to the toxicities, biases and ignorance of worldwide users. While we remain braced for challenging exchanges, as reflected in our co-written constitution, the creation of a partially enclosed app-based digital space in Discord allows members to feel unplugged from the exposure of a site like Twitter, in turn enabling safer and more open interaction (Creechan et al., 2021).

As we began to grow, we realised we needed to reassess who the network is for and who its beneficiaries are. Having started within academic strictures, we recognised the unethical and appropriative stakes of discussing neurodiversity without broader community input. As neurodiversity studies is developing as a critical field, the academic membership needed to engage with the paradox of advocating for “diversity” while being complicit in a system that privileges the perspectives of white, middle-class, autistic academics from the Global North, who have had access to formal diagnosis (Betilsdotter Rosqvist et al., 2020b). As such, we broadened the purview of the network to include anyone with an interest in the relationship between neurodiversity and narrative. Some of our most active members are situated outside of the academy but are fully engaged in current scholarship and research conversations on the Discord server. One such member explained:

As a person who isn’t a traditional academic and who doesn’t have access to academic discourse, the network means I no longer feel isolated in my interests.

Similarly, another member described the space as “a bit of an online oasis” and reflected on their ease within a space where being neurodivergent is a majority position. When establishing the network, we decided not to limit membership to individuals who identified as neurodivergent or to doubt the validity of self-diagnosis. It was agreed that, so long as the network remained neurodivergent-led (in practice, a consistent and significant majority of neurodivergent administrators) and inclusive for our neurodivergent majority, those who identified as neurotypical were welcome to contribute to the server. There was a slight concern about the need to negotiate a means of “cross-neurotype communication” (Hillary, 2020a) when we decided to open the space to neurotypical allies, but we found that by actively decentering the expected norms of online communication, we mitigated the need for performative neuronormativity and, as such, our neurotypical members learned to respect the flexibility of our space and there was no need to institute additional supportive measures or “translation” practices for those unused to being in the neurological minority. It was striking that when asked about the network, members commonly referred to the space as “affirming” and “freeing,” as the neurodivergent majority meant members could “think through ideas without the pressure of the “NT gaze” of academia” (McDermott, 2022). Indeed, others commented that, within the network, academic ideas could be articulated according to communication preference, a rarity in conventional academic spaces. One member commented that they “feel more articulate in text, compared to in-person,” a communicative choice that is easily supported by the server (Donaldson et al., 2021). Time and again, members positioned the network in opposition to the academy and to traditional models of scholarship and knowledge production. It was particularly striking that network discussions could cover the same material, but that they were perceived as different and, as such, liberating—as one member observed:

General and academic contributions in this space feel a lot more relaxed for me because of the inclusive social expectations—I don’t feel nearly as self-conscious about stuff like rambling, overapologizing, bringing up things that the conversation has moved on from etc. At the same time, I feel like there’s a bit of a gap where I have to consciously stop self-policing by neuronormative standards? Like if I want to keep popping on/off and/or keep my camera off in a call I feel the urge to apologise or power through the fatigue and just stay on and then I have to remind myself that people in this space probably don’t care that my camera is off.

Some of us have expressed loss and frustration that, having been taught to manage our neurodivergence for neuro-majority spaces, traditional educational systems had affected and even stifled our ability to think and express ourselves naturally (Freire, 1970; Wood, 2019). We lament the loss of creative and intellectual potentialities offered by our divergences that we could have harnessed had we been encouraged to embrace them.

This article celebrates the creative and emancipatory possibilities of an online neurodivergent-majority space that prioritises peer support, mutual aid, and community formation. As the pandemic mainstreamed remote collaboration to an unprecedented level, we realised the possibilities an online space could offer neurodivergent individuals outside the oppressive and pathologising strictures of societal institutions, including the classroom, the courtroom, the psychiatrist’s chair, and the academy. The network will continue to evolve as our understanding of one another and what it means to be neurodivergent develop. Within the social sciences, discussions of neurological differences actively resist the dominant medical framings of neurodivergence, which has led to important discussions of harms perpetuated by dehumanising research practices (Pellicano & den Houting, 2022). Nonetheless, to focus solely on the impact of institutions and social structures on neurodivergent persons situates us only as subjects and/or victims of neurotypical society or of our own neurological difference. By recognising the power of narrative as a means of exploring our differences, we have found an alternative, empowering approach through which we can connect with one another and advocate for ourselves. Each strand of our regular network activities foregrounds narrative and storytelling: our discussions of fictional narratives in our monthly mixed-media reading/watching groups, our “show and tell” sessions, our work-in-progress research discussions, and the sharing of thoughts and opinions on Discord forums are all mediated through narrative. This mosaic of narratives enables us not only to celebrate the intellectual outputs of our members but also to share in pleasurable activities and hobbies they enjoy, supposed frivolities which are so often discouraged or dismissed by capitalist dynamics that focus resolutely on productivity and use-values. Instead, we believe in the value of personal narratives. By cultivating a space to share these narratives, we have established a community of care through which we can begin to tackle epistemic injustice (Chapman & Carel, 2022).

## 3 Narratives of Neurodiversity

Our early identity as the Neurodiversity and Literature Network, which had connotations of specialist (and therefore exclusionary) forms of writing, was soon changed to the NNN. This shift allowed us to conceive of literature more broadly, encompassing all sorts of stories about neurodiversity, from various perspectives and in myriad formats. The shift from “literature” to “narratives” signified a sort of plurality, where neurodiversity could be recognised and discussed beyond mainstream or canonical representations, allowing alternative writing formats such as blogs, social media posts, zines, and pamphlets. Recognizing our members’ varied access needs, we were inspired by this broadened focus to extend beyond written formats to include performance art, film, television, podcasts, and stand-up comedy. The discussion of these narratives would become integral to the network’s community focus, but formal consideration also inspired us to conceive of the content published within the group—posts, comments, and dialogues—as narratives in their own right that signalled a burgeoning neurodivergent counterculture. In this way, “narrative” came to be understood within the network as involved in the active construction of new stories and modes of articulation through which we can engage with identities and experiences that were previously marginalised in representations aimed at the neuro-majority.

The shift from the conjunctive “and” of our original name to the prepositive “of” also signalled the network had become a space for exploring how neurodiversity itself is narrativised both culturally and as a lived experience. Many of our members are acutely aware of the stories constructed through the medical model of disability, where neurological difference has been figured as a “disordered” way-of-being in need of mitigation, change, or eradication. After all, what is a list of symptoms, if not a form of narrative? This predominant rhetoric continues to proliferate through public consciousness, manifesting as stigma and discrimination, and subsequently placing neurodivergent persons under intense scrutiny and impossible pressures. As a reaction in part to the strictures of the medical model, the neurodiversity movement has typically understood neurodivergence through the social model of disability, which posits that it is not the neurological difference that constructs disability, but ableist social structures (Oliver, 1983). The social model, however popularised, has its limitations, is often invoked to focus on structural failing, and thereby downplays the physiological and cognitive elements that create challenges no matter the structure of the wider society. We believe that narrative offers a third model for thinking about neurodivergence. Narrative gives credence to the expertise of lived experience and resituates the neurodivergent person as the subject, as opposed to the object, of the model. We refer, in particular, to any narratives that unsettle the dichotomization of human perspectives into cognitive types or discrete discourse communities that conform to existing networks of power (Yergeau, 2010). We are particularly interested in those stories—personal, collective, or speculative, which convey agency, rather than passivity and subjugation, upon those who are regarded as neurologically “atypical.” As one member, who is a SEN educator, comments:

There’s a huge clash between the pathology and neurodiversity paradigms/narratives…and one of the ways in which the neurodiversity paradigm is making headway (this is in itself a narrative element!) is through the critical exploration of narratives of many types. Ugh, I didn’t express any of that well, but I hope I am conveying the idea of living in and with a bunch of narratives and also seeing narratives as having the potential to change education for the better. So narratives are both the what and the how??

For this reason, our network functions as a long-form, open-ended and non-hierarchical arena to explore the nuances of our own narratives via interpersonal communication and engagement with an array of media. Relatedly, adding a proactive “creative writing” channel helped foster a culture of narrative-making for members keen to express or explore their stories through their own craft, while also providing a space for the mindful and escapist decompression creativity can enable.

But, what are these narratives specifically? And, why is storytelling so important to neurodivergent people? Contrary to common medical-model assumptions, neurodivergent readers empathise, take perspectives, and participate in communal thinking in reading stories (Chapple et al., 2021). The successes of contemporary neurodivergent authors, such as Elle McNicoll, Katherine May, Joanne Limburg, and Rivers Solomon, have been the subject of network discussions and are deemed to actively demonstrate the value and possibility of neurodivergent storytelling. When we invited our members to respond to the questions “what do stories give us?” and “how do stories enable us to think about our neurodivergence?” responses were often interwoven with concerns around identity formation. Stories give us “reality,” writes one member, who refines this definition as “a consensus story; the story we, they, or you tell ourselves about ourselves.” Narratives can both offer empowerment and expose abuses of power. One member describes how their autism and ADHD diagnoses were insidiously “predicated on the idea— literally in the diagnostic report—that I have no imagination and can’t create stories or think creatively.” These diagnostic reports are themselves narratives, relying on an authoritative medical rhetoric in their (increasingly futile) attempts to define and categorise neurodivergence. One member notes the importance of following diagnostic shifts, positioned as:

A story about the conflict between clinic and constitution: Who defines what we are made up of? How do we define what we are made up of? What mediums and expressions allow us to write the stories that ensure our cultural, economic, political, and social freedom to define our value on our own terms?

Within the context of these wider societal pressures, allowing neurodivergent persons to narrativise as a mode of creation or self-exploration becomes a political imperative and, for some, engagement with stories and storytelling can be a means of self-care. As one member notes, writing stories “has saved my life on more than one occasion. I know I am not mentally well when I can’t read or write. I write fiction and non-fiction, to help me process information and explore my feelings.” Academic discourse suggests stories can place too many communicative demands on neurodivergent individuals to have a wholly positive effect on wellbeing, but through discussions with the network, we are inclined to agree with Hilde Lindemann Nelson’s assessment that narratives can be good for us insofar as they allow us to create new counter-stories that reject normative understandings of our identities (Nelson, 2000). By foregrounding narrative exploration *and* creation, the network offers a space where storytelling is valued as a fundamental element of neurodivergent lived experience rather than an activity seen as beyond the capacity of those with certain neurodivergent diagnoses.

## 4 Narratives of Identity

Personally, neurodivergence is itself a story. As a story, I have a sceptical relationship with it and I’m more than aware it’s a story of contestation.

Organically, our focus on fictional narrative became enmeshed with discourses of self-exploration (Hillary, 2020b). We see this tendency as an ironic and empowering reversal of the neurotypical tendency to “story” autistic (and other neurodivergent) persons that Yergeau (2018) observes in *Authoring Autism*. New members introducing themselves often explain they are joining partially to make sense of their neurodivergence. As such, we recognise peer support and shared experience as important values for self-understanding (Rose, 2005). Exchanges between members provide respite from dominant cultural scripts that typically align with medical or pathological interpretations of neurological difference. Here, they find empowering terms, concepts, and stories that support a positive and an affirming sense of self that embraces their neurodivergence. As one member puts it, the network grants us tools and resources to “learn *with* each other” rather than from each other, as we continue to develop our individual and collective understanding of our respective identities. Indeed, members frequently remark that the network fosters thinking-with and feels like a site of thinking-together which, in Erin Manning’s words, allows “a coming into itself of thought through a coming out of it-self of the individual” (Manning, 2020, p. 7). One member’s introductory post read:

I’m a second-year social anthropology undergraduate who only recently (a few months ago) realised I was neurodivergent. I haven’t been formally diagnosed and have decided not to seek diagnosis but think that I now understand myself better and find it really affirming to connect with other ND people.

These reflections are common on the server as many members use the space to explore their respective diagnoses or challenge the categorisation of these criteria. That said, some members find these diagnostic narratives helpful, but only once reconfigured by their own thinking: “For me, I prefer autistic and allistic as terms; you’re either autistic or you’re not”; they elaborate to say that the creation of this autistic/allistic binary enables autistic persons to create an empowering independence from the equalising agenda of the wider neurodiversity movement where their specific needs could be negated. Other members express dissatisfaction with diagnostic labels as they find them limiting and totalising. “Being someone who doesn’t see themselves as neurotypical I identify with autism rather than as autistic (I consider myself non autistic),” one member writes, “I appreciate how [the] celebration of our uniqueness doesn’t depend on labels or diagnostic status [on the server].” This member’s hesitancy to identify as autistic is predicated more on their “fundamental issue with the idea of neurotypicality” which they consider a similarly constructed social narrative. Their comfort in “a sense of familiarity with certain aspects of neurodivergence” demonstrates how self-narrativization empowers, a conclusion reached through conversations and reflections within the network space. Here, members create shared understandings—even if not shared by everyone; for instance, in response to the question “what does it feel like to be part of the network?” one member joked about the “joys of alexithymia” and, in reaction, another member gave their response to the same question as “still alexithymic!” This shared understanding of aspects of neurodivergence can create humorous inter-relations while establishing feelings of belonging and community (Bertilsdotter Rosqvist, 2012).

Although neurodiversity is an empowering concept in many ways, it is nevertheless mired in the values and knowledge practices of the Global North and the centring of anglophone constructs, meaning that some narratives of neurodiversity are better represented than others both within the network and in culture more broadly. This poses challenges for those whose identities do not conform to these dominant paradigms, and the individualist and cognitivist values that they impart. As it stands, neurodiversity remains Global North-centric as the disparity of published works on the subject from the Global South attests. One member, who is Tagálog, explains that while she identifies with the aims and values of the Network, she is still working out how neurodiversity harmonises or intersects with other aspects of her identity:

It’s still unclear to me how I can integrate my autistic identity with my Tagalog identity. I don’t even know how to translate or if I can even translate how being autistic is in Tagálog. It’s not a matter of disliking that the emancipatory concept of being autistic came from the Global North. I accept it and I acknowledge how it’s now one of the conceptual tools that help me understand myself. I just feel at a loss on how to relate this with my local identity that won’t erase my Tagálog identity. English/American influence already did so much damage to our collective Tagalog identities here in the Philippines. I do hope this network can connect me with people who can relate with these issues in their local contexts.

This comment reminds us that the neurodiversity movement was not birthed in a vacuum, and is inextricable from the social, cultural, and political contexts of its emergence. While neurodiversity is a cross-culturally salient *human* phenomenon, the neurodiversity movement is fundamentally grounded in Western Europe and North America. Frameworks, terms, and schemas that dominate discussions (especially diagnostic terms) may be oblivious to the contexts in the Global South and can be awkward superimpositions onto unique and incommensurable ways of understanding human diversity. Neoliberalism, colonialism, and racism have not just shaped hegemonic ideas of the “normal” or the “neurotypical,” they have also interacted in multidimensional and frequently violent ways with Global Southern approaches to neurodivergence that are often grounded in local spiritualities. While the deep and profound influence of culture in shaping voice-hearing experiences has now been well-established (Luhrmann & Marrow, 2016), there is a striking gap on research that explores how other forms of neurodivergence in the Global South shape unique subjectivities and forms of belonging. As a collective involved in discourses regarding the future of neurodiversity, we must acknowledge our biases, limitations, and contexts. For example, we must recognise that when we talk about challenges of being neurodivergent, we tend to imagine that these challenges occur only within our majority geopolitical and cultural context (in the case of the network, there is a large UK majority), even if they are more widely applicable. As a member writing from “an urban and privileged context in India” explains: “When it came to being diagnosed with ADHD, dyslexia, autism, etc., in an educational context it really wasn’t pursued unless you were a ‘problem’ kid.” While there are some parallels here across countries and continents, the assumed point of comparison is situated within the contexts of the Global North. While it is possible for some members to identify themselves as being part of the network, cultural nuances and Western biases mean that their perspectives are not always represented under the “us” of our collective identity. Shaping a group identity means recognising plurality within a shared social space, placing emphasis on difference, paying attention to our social, cultural, and political situatedness, and acknowledging that some of us have better access to cultural narratives of neurodiversity than others. While the network is a space for those with similar experiences and shared social identities to come together, we are inevitably implicated in the broader systems we aim to resist and must therefore be mindful of the potentially exclusionary natures of the narratives we share to actively commit to cultivating a more intersectional space. For example, the scheduling of our live Salon discussions tends to align with the availability and time zones of the UK-based administrators who most often coordinate them. This adherence to European norms arguably exerts an exclusionary structural influence on non-Europe-based members that requires addressing.

## 5 Fostering Care Amidst Austerity and the Pandemic

While we have tried to develop an inclusive, neurodivergent-led online community, administrators have sought to understand their responsibilities to the members and each other as we recognise the disproportionate effects of austerity and the pandemic on disabled people. For this reason, we have compiled support materials that connect to our broader “networks” of care.

As many of us are also educators, we also recognised our privilege in accessing texts, spaces and social networks others are excluded from on the basis of their perceived identities and capacities. However, Discord has itself served as a “facilitator” that enables us, admins, to participate in the network as equals, and to disclose our own narratives of neurodiversity. Although it is a digital server, it also makes human connections possible, with one member writing about its “potential for collaborative work, esp. in the vein of academic discussion, news and current affairs vis-à-vis neurodivergence.” As this comment suggests, the app itself is an extension of our community, enabling new stories and relations to form between those who would not be able to connect in the same way without it. It is also an affective space, where we can share not only words and fully formed judgements but images that half-formed ideas that excite us or perplex us, frustrate us, or which just puzzle us. As in the real world, there is often a sense of “not knowing,” which makes it quite different to “academic space.”

Unlike many platforms that we engage with in our lives, Discord was not developed to improve productivity in education or work. Discord was instead designed to connect players of online games around the world. Zoom offered a free peer-to-peer communications tool that did not require an institutional account, at least for shorter meetings. Both channels provide a greater range of non-speech expressive tools via emoticons and handgesture symbols than many other platforms. The multiple chat channels on Discord remain live after meetings, allowing individuals to communicate at a pace that suits them, and to loop back to ideas introduced six months ago. There is no need to time-limit questions or answers, nor to see any answer as definitive. So, while intimacy is often assumed to be something produced within private as opposed to community spaces, we discovered the beginnings of real friendships through the server. At the same time, however, we do not see the Network as a utopian project because the connections we have facilitated have no doubt excluded some. For instance, we often rely on academic language and we rely on technology that is inaccessible to some. While some have adjusted to its format, some people still find Discord “too busy” and/or overwhelming.

Equally, we recognise we could not have imagined the network space’s possibility without the affective, intellectual, and creative labour of the members. As feminist critics of science and technology studies demonstrate, the connections and exchanges fostered by these forms of networks are typically seen as too “subjective” to constitute the basis of knowledge (Latimer & López Gómez, 2019). Some see caring about those we work with and think alongside as introducing bias. They claim our ties to each other may obscure the supposedly “objective” view typically expected in academic knowledge production. Yet what we are studying, as literary critics or cultural theorists, is produced by people and through socially enacted material arrangements. As much as we are responsible to others for the work we produce—many of us aim to flourish as a means of advocacy for neurominority people—we need to be open to the vulnerability of being challenged and changed by others, and to be transparent in our communicative acts. We may struggle to participate in the spaces dictated by our professional status, or we may find ourselves able to access certain spaces only if we mask our differences and access needs. Technological mediation does not, contrary to conventional ideological constructions of “individuals” and “relations,” inhibit either intimacy or autonomy. The network instead provides energy and opportunity to pursue our work within an environment somewhat closer to equality. This article, therefore, offers a space to consider the affective constraints on our own ethical practices and the power relations within which we are enmeshed.

## 6 Afterword

To reflect on the article’s collaborative production, this brief afterword details the access considerations made during the process. Tasks, such as refining research questions, inviting and collating network responses, writing, and copyediting, were divided according to strengths and expertise; for instance, one dyslexic co-founder struggles with writing from a blank page, so was responsible for re-drafting and later edits. Scholars from the Global South had editorial control over the sections discussing the eurocentrism of the neurodiversity movement. Regarding our approach, feedback from members has been overwhelmingly positive, as one comments:

I like that we were given the opportunity via draft feedback to qualify the answers we gave to the questions and also understand the specific context in which our words will be going out (which can be a source of special anxiety to many ND folk, I think). Also helped reinforce the sense of being research collaborators not subjects.

We wanted to make each stage of the publication process as transparent and as participatory as possible, but we found that aspects of the academic publication process made this ethos more difficult to follow. When it came to the peer-review, one reviewer pointed to the irony that the review process is not quite as collaborative as our working method. In order to facilitate a collaborative revisions process, we gave editing privileges to all network members and asked that the track changes function was used to identify small textual amendments and that the comment function was used to ask broader questions or for larger points of clarification that had the potential to change the direction of the overarching discussion. We used an additional document to present the reviewer feedback to the network and for members to assign themselves to revisions that intersected with their personal investment in the discussion; this document then became the response to our reviewers. Where revisions prompted significant further discussion, members were invited to share their thoughts on our Discord server, and we used the forum function to untangle ideas. Although it may seem oddly utopian, we experienced no significant difficulties encountered during the co-writing process. We have reflected upon this relative ease, and we think that the inter-personal relationships that we had developed through previous network activities, our commitment to discussing our respective needs, and our willingness to place equal value on contradictory views may have contributed to the level of positivity experienced by our members in the preparation of this article. We hope that we have managed to capture the nuances, understandings, and collective empowerment that we have felt through our continuing pandemic (and future) project, the NNN.
